# Cost-Effective Surgical Management of Pterygium: A Blood-Based Adhesion Technique Substituting Fibrin Glue

**DOI:** 10.7759/cureus.57786

**Published:** 2024-04-07

**Authors:** Audrey Yan, Ryan Meng, Casey O'Doherty, Leo Wan, Ramakumar N Gounder

**Affiliations:** 1 Department of Medicine, West Virginia School of Osteopathic Medicine, Lewisburg, USA; 2 Department of Ophthalmology, Global Ophthalmology, Moon Township, USA

**Keywords:** pterygium surgery, eye surgery, ophthalmology, cornea, pterygium, ophthalmologic surgery

## Abstract

Pterygium is a degenerative eye condition marked by the abnormal growth of conjunctival tissue over the cornea, primarily affecting individuals near the equator. When it reaches the cornea’s center, patients may experience obstructed and blurry vision, necessitating pterygium surgery. The standard surgical approach involves excision with a blade, using a conjunctival autograft to address the defect, and securing it with fibrin glue. Recurrence rates exhibit variability, with approximately half occurring within the initial three months. In this case, we present a more cost-effective surgical approach, avoiding the use of a blade to minimize intraoperative complications. Additionally, autologous blood is employed instead of fibrin glue. We evaluate immediate and post-operative complications, as well as the incidence of recurrence rates at the three-month mark.

## Introduction

Pterygium is a degenerative eye condition marked by the abnormal growth of conjunctival tissue over the cornea, resulting in the development of triangular-shaped fibrovascular tissue. The primary risk factor associated with the development of pterygium, also known as *surfer’s eye*, is prolonged exposure to ultraviolet (UV) radiation [1]. Other factors include lower income, older age, irritants such as dust and wind, male gender, smoking, and genetic predisposition. Pterygium is prevalent globally, but a higher incidence is seen in regions located about 30 degrees to the north and south of the equator [2]. Symptoms of pterygium include a foreign body sensation, redness, ocular burning, and astigmatism. In cases where the pterygium extends to the central part of the cornea, patients may present with blurry vision [1]. When the impact on visual acuity is minimal, initial treatment involves the use of topical eye lubricants. However, surgical resection often becomes necessary if the pterygium starts affecting visual acuity or if the patient expresses cosmetic inconvenience [3].

Surgical treatment for pterygium is categorized based on the technique used to manage the defect after excision. The eye is typically prepared by applying Xylocaine® jelly or subconjunctival 2% lidocaine with 1:100,000 epinephrine. The pterygium is then excised using a surgical blade. Options for managing the resulting defect include leaving it exposed through bare sclera excision or covering it with the surrounding conjunctiva via primary closure. Alternatively, a flap of tissue can be utilized through a pedicle flap or the pterygium head can be transposed to cover the defect [4]. Tissue grafts can also be utilized through a conjunctival autograft (with or without limbus) or amniotic membrane graft [5]. Other tissue sources include lamellar keratoplasty and buccal mucous membrane grafts [6]. Additional treatments include laser procedures such as yttrium-aluminum-garnet (YAG) and a polishing technique developed by Barraquer [7-8]. Recurrence rates can vary, but in most studies, 90% of recurrences occur between the first and third month [9].

Simple excision techniques, which involve removing the pterygium and leaving the sclera exposed, often result in higher recurrence rates. However, combining pterygium excision with a tissue graft significantly reduces the risk of recurrence [10]. This method, first introduced in the 1980s, involves the removal of adjacent conjunctival tissue from the patient’s eye to cover the excised pterygium area. Depending on the severity of the pterygium and surgical preference, the autograft may include the use of limbic tissue located at the border of the cornea and sclera. This limbal tissue contains stem cells that maintain the health of the cornea. Various methods can be employed to connect the autograft to the host site, including traditional approaches such as sutures and fibrin glue, as well as newer methods like the use of autologous blood [10,5]. In this case presentation, pterygium surgery was conducted without employing a surgical blade for excision and utilizing autologous blood as the graft material.

## Case presentation

**Figure 1:**
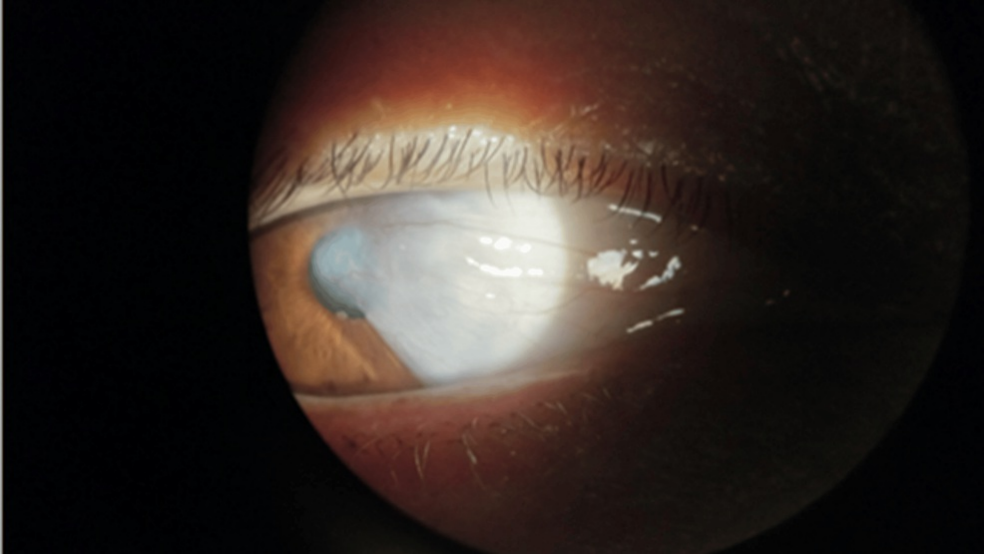
Nasal pterygium encroaching upon the central visual axis of the right eye.

A 48-year-old female with no significant medical history presented to the clinic for persistent blurry vision, steadily worsening over the years. The patient was an immigrant from Mexico working as a hotel cleaner, accompanied by her son due to a language barrier. Upon examination, a progressive nasal pterygium approaching the central visual axis of the right eye was observed (Figure 1). While her left eye demonstrated 20/20 vision on the Snellen eye chart, her right eye exhibited 20/200 vision with astigmatism. After a thorough discussion, including explanations of the procedure, potential risks, benefits, expected outcomes, and alternative options, the patient consented to undergo the surgery, and the procedure was scheduled. The patient was unable to afford medical insurance and covered the expenses out of pocket. Taking this into consideration, the patient was allotted a maximum of 30 minutes in the operating room.

The pterygium was identified, and the base was marked with a marking pen and calipers. Subconjunctival 2% lidocaine with 1:100,000 epinephrine in a syringe with a 30-gauge needle was then injected under the pterygium. A radical incision was made around the body of the pterygium using Westcott scissors and 0.12 forceps, in place of a standard blade. The 0.12 forceps were used to turn the eye inferior to mark the donor graft site at the superotemporal conjunctiva. A graft site was measured and outlined with an ocular-safe marking pen. Subconjunctival 2% lidocaine with 1:100,000 epinephrine was then injected under the marked conjunctival area. The conjunctival autograft was then excised using Westcott scissors and bent tying forceps. Bent-tying forceps were then used to remove the head of the pterygium. After the entirety of the pterygium was removed, any fibrous tissue was excised. A #11 blade was used to smooth the corneal defect, limbal bed, and scleral bed. The graft was transposed to the pterygium excision site with extra care taken to place limbus to limbus on the scleral bed. Once the graft was secured with pressure from two round tip swabs, the antibiotic ointment was applied over the right eye, and the patient was transferred to the postanesthesia care unit in stable condition. Before patching, a bandage contact was placed on her eye.

Postoperative outcomes

**Figure 2:**
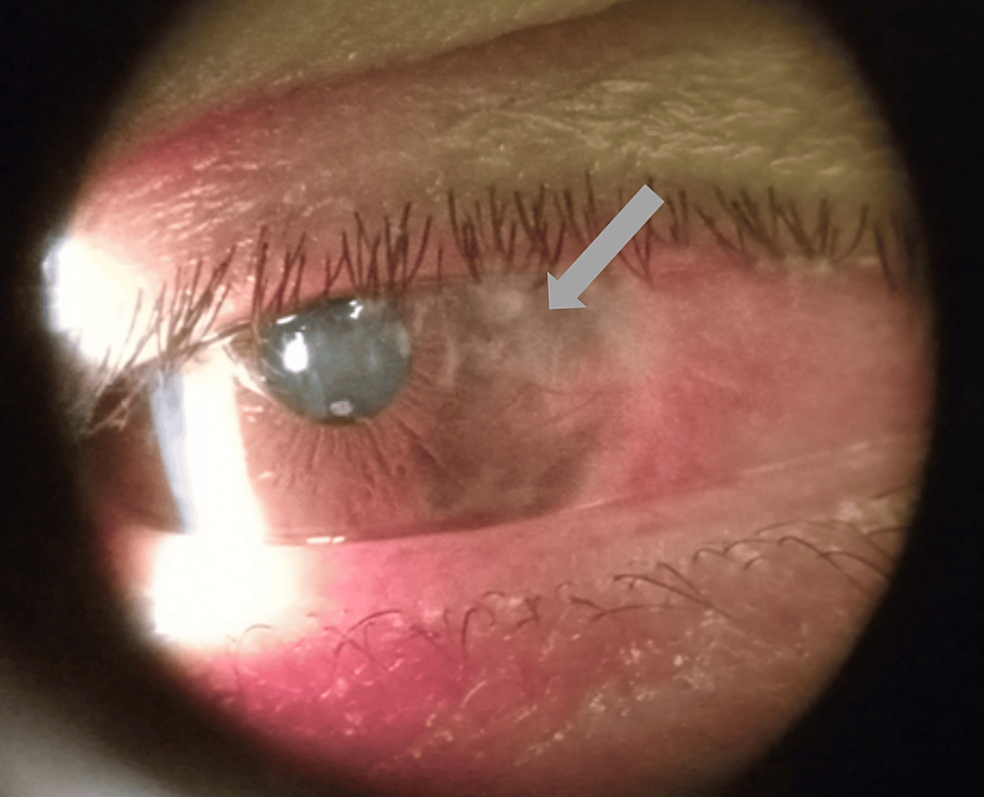
Exposure of Tenon’s capsule, right eye.

The patient revisited the eye clinic three days later with no significant complaints other than mild itchiness. Visual acuity testing revealed 20/60 in the right eye, which improved from 20/200 at the initial consultation. Extraocular movements, including the medial rectus muscle, remain intact. However, upon examination, it was not completely clear if the graft was still there. The patient was prescribed Neomycin/Polymyxin B/Dexamethasone (Maxitrol) ointment and Ivizia suspension. During a two-week follow-up, the patient continued to do well, reporting no pain. Upon examination, it was confirmed that the graft was lost, most likely during the immediate postoperative period. Additionally, it was observed that the conjunctival tissue has not fully grown over Tenon’s capsule, displaying a red/brown color (Figure 2). The patient was restarted on Neomycin/Polymyxin B/Dexamethasone (Maxitrol) ointment nightly and Ivizia drops twice a day.

**Figure 3:**
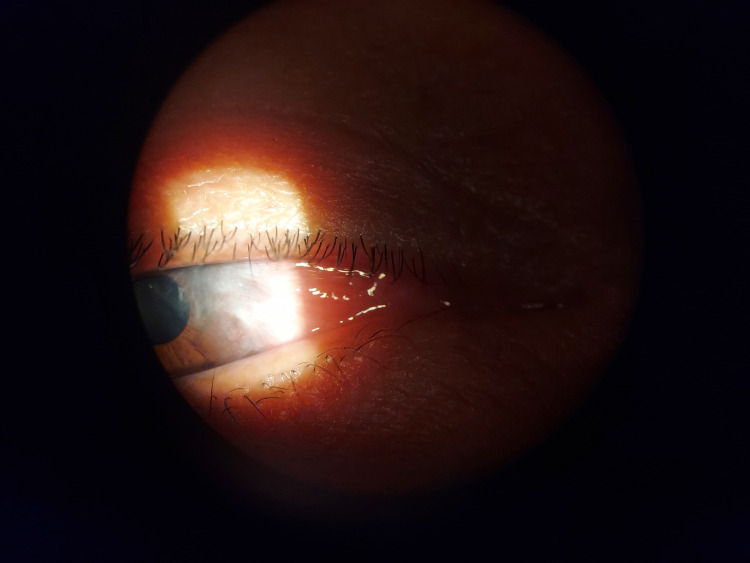
Central cornea clear, right eye.

The patient returned three months later, overall doing well but still expressing some irritation. The eye exhibited reduced redness, and the central cornea was clear (Figure 3). Visual acuity was slightly improved at 20/50 in the right eye with high patient satisfaction. In response to the irritation, the patient was given artificial tears.

## Discussion

Pterygium is most common in individuals who live 30 degrees north or south of the equator, which includes those from Brazil, Saudi Arabia, Namibia, Australia, Chile, Egypt, and Mexico. The patient, a Mexican migrant who works as a hotel cleaner, was at higher risk since she was frequently exposed to dust and toxic chemicals. The goal of pterygium surgery is to restore ocular surface regularity, preserve visual acuity, and avoid recurrence. Nevertheless, there are possible side effects from the frequently performed pterygium excision with conjunctival autograft surgery, including intraoperative risks such as globe perforation, scleral or corneal thinning, bleeding, muscle injury, and graft tear. Early postoperative complications may involve corneal dellen, subconjunctival hemorrhage, graft edema, graft loss, and granuloma, while late postoperative complications include recurrence, scleritis, and endophthalmitis [11].

In traditional pterygium surgery, a blade is used for precision, and fibrin glue is used to secure the autograft closure. Fibrin glue can reduce surgical time and lessen postoperative inflammation, reducing the need for corticosteroid therapy and improving patient comfort [1]. Although fibrin glue improves security, those without health insurance may find treatment prohibitively expensive - between $600 and $700. Additionally, there are some dangers associated with improper usage of a blade, such as scleral or corneal thinning [12]. This emphasizes the importance of considering the socioeconomic status of individuals undergoing such procedures. The patient was unable to afford medical insurance, making surgery expensive, especially if complications arose with the use of a blade. Appealing alternatives include lower risk, cost-effective methods such as the presented method of using autologous blood as a tissue adhesive for graft fixation. Studies have shown promising outcomes with this technique, boasting low rates of complications such as granuloma, conjunctival hemorrhage, and graft dehiscence [13]. Moreover, the relatively low recurrence rate, 2%, and improvement in visual acuity observed postoperatively further validate the efficacy and economic viability of this approach [13]. Therefore, using autologous blood is a safe, effective, and economical procedure with less discomfort and more patient satisfaction.

Following surgery, our patient recovered well, reporting only mild irritation. The graft loss observed two days later could be attributed to the absence of fibrin glue for tight security. Despite this, the patient remains content with relatively good vision.

Although recurrent pterygium may appear many years later, the majority of recurrences happen during the first three months [9]. Higher rates of graft loss were observed using autologous blood in a study evaluating several procedures, including the use of fibrin glue, autologous blood, and/or sutures. All three methods, however, exhibited low rates of recurrence, suggesting that each treatment has provided good outcomes for patients [12]. Choosing not to use a blade may result in better outcomes for both the surgeon, in terms of ease, and the patient, as alternative techniques are less likely to cause eye damage. The use of fibrin glue may be more essential to prevent graft loss but should still be considered, particularly for those facing financial challenges, given its still low recurrent rate. This study cannot conclusively determine whether recurrence will occur beyond three months.

Furthermore, the standard duration for pterygium surgery is typically between 30 minutes and an hour [4]. However, considering the patient's out-of-pocket payment and the desire for cost-effectiveness, we decided to allocate only 30 minutes for the surgery. By aiming for a successful procedure with a recurrence rate within typical ranges, this approach not only minimizes costs but also streamlines care delivery, ensuring efficient use of resources and timely treatment for the patient.

Subconjunctival anesthesia, administered either as 2% lidocaine with 1:100,000 epinephrine or Xylocaine® jelly, is commonly used in pterygium surgery, as seen in this case. However, it is important to note that the injection process can be uncomfortable for the patient. While needle injection is effective, it comes with potential complications such as hemorrhage, conjunctival injection holes, and globe perforation, which were fortunately not observed in this case [14]. Recent studies suggest that lidocaine 2% gel is a viable alternative, providing sufficient anesthesia and improving patient comfort. This gel also offers extended contact time with the ocular surface, ensuring prolonged lidocaine release and effective lubrication of the cornea, reducing the need for frequent wetting. This method can be a preferred choice for both patients and surgeons, especially when patients fear injections or demonstrate poor cooperation during surgery. 

Given that pterygium primarily affects regions around the equator, which are often underserved in terms of healthcare access, there is a possibility that physicians may be able to swiftly remove pterygiums, similar to how cataract surgeries were traditionally performed. This expedited approach could be particularly advantageous in areas with limited resources, allowing for more efficient treatment delivery to a larger number of patients in need. The combined potential of xylocaine jelly with the demonstrated method of bladeless pterygium excision with autologous blood autograft allows for an overall minimally invasive and highly cost-effective method of pterygium removal while maintaining a high degree of patient satisfaction.

## Conclusions

Pterygium surgery may be necessary for individuals residing close to the equator. Physicians in the United States must stay informed about both traditional and alternative methods of pterygium surgery, especially as immigration rates increase in the country. This case report explores a financially favorable procedure that eliminates the use of a blade to mitigate potential eye complications and avoids the use of expensive fibrin glue. Despite observing graft loss, possibly attributed to a lack of fibrin glue, this case report suggests that the recurrence rate in this surgical procedure may remain low compared to typical recurrence rates. This procedure should be considered as an option for patients facing financial constraints.
